# Analyzing and Forecasting Pediatric Fever Clinic Visits in High Frequency Using Ensemble Time-Series Methods After the COVID-19 Pandemic in Hangzhou, China: Retrospective Study

**DOI:** 10.2196/45846

**Published:** 2023-09-20

**Authors:** Wang Zhang, Zhu Zhu, Yonggen Zhao, Zheming Li, Lingdong Chen, Jian Huang, Jing Li, Gang Yu

**Affiliations:** 1 Department of Data and Information Children's Hospital, Zhejiang University School of Medicine Hangzhou China; 2 Sino-Finland Joint AI Laboratory for Child Health of Zhejiang Province Hangzhou China; 3 National Clinical Research Center for Child Health Hangzhou China

**Keywords:** time-series forecasting, outpatient visits, hospital management, pediatric fever clinic, long sequence, visits in high frequency, COVID-19

## Abstract

**Background:**

The COVID-19 pandemic has significantly altered the global health and medical landscape. In response to the outbreak, Chinese hospitals have established 24-hour fever clinics to serve patients with COVID-19. The emergence of these clinics and the impact of successive epidemics have led to a surge in visits, placing pressure on hospital resource allocation and scheduling. Therefore, accurate prediction of outpatient visits is essential for informed decision-making in hospital management.

**Objective:**

Hourly visits to fever clinics can be characterized as a long-sequence time series in high frequency, which also exhibits distinct patterns due to the particularity of pediatric treatment behavior in an epidemic context. This study aimed to build models to forecast fever clinic visit with outstanding prediction accuracy and robust generalization in forecast horizons. In addition, this study hopes to provide a research paradigm for time-series forecasting problems, which involves an exploratory analysis revealing data patterns before model development.

**Methods:**

An exploratory analysis, including graphical analysis, autocorrelation analysis, and seasonal-trend decomposition, was conducted to reveal the seasonality and structural patterns of the retrospective fever clinic visit data. The data were found to exhibit multiseasonality and nonlinearity. On the basis of these results, an ensemble of time-series analysis methods, including individual models and their combinations, was validated on the data set. Root mean square error and mean absolute error were used as accuracy metrics, with the cross-validation of rolling forecasting origin conducted across different forecast horizons.

**Results:**

Hybrid models generally outperformed individual models across most forecast horizons. A novel model combination, the hybrid neural network autoregressive (NNAR)-seasonal and trend decomposition using Loess forecasting (STLF), was identified as the optimal model for our forecasting task, with the best performance in all accuracy metrics (root mean square error=20.1, mean absolute error=14.3) for the 15-days-ahead forecasts and an overall advantage for forecast horizons that were 1 to 30 days ahead.

**Conclusions:**

Although forecast accuracy tends to decline with an increasing forecast horizon, the hybrid NNAR-STLF model is applicable for short-, medium-, and long-term forecasts owing to its ability to fit multiseasonality (captured by the STLF component) and nonlinearity (captured by the NNAR component). The model identified in this study is also applicable to hospitals in other regions with similar epidemic outpatient configurations or forecasting tasks whose data conform to long-sequence time series in high frequency exhibiting multiseasonal and nonlinear patterns. However, as external variables and disruptive events were not accounted for, the model performance declined slightly following changes in the COVID-19 containment policy in China. Future work may seek to improve accuracy by incorporating external variables that characterize moving events or other factors as well as by adding data from different organizations to enhance algorithm generalization.

## Introduction

### Background

COVID-19 is the most severe global pandemic of the 21st century, which has brought major changes to the global health care environment [1]. According to statistics from the World Health Organization, there have been >760 million confirmed cases of COVID-19 worldwide, including nearly 7 million deaths to date. Although the World Health Organization has declared that COVID-19 no longer constitutes a “public health emergency of international concern,” it remains a serious infectious disease that will persist for the foreseeable future. Moreover, since the onset of the epidemic, numerous epidemic infections have also emerged, including influenza A, respiratory syncytial virus infection, and mycoplasma pneumonia. Successive waves of respiratory infections led to a significant increase in the number of patients presenting with fever. This prompted governments and hospitals to take measures for patient management to prevent viral transmission and control the risk of hospital-acquired infections [2,3].

**Figure 1:**
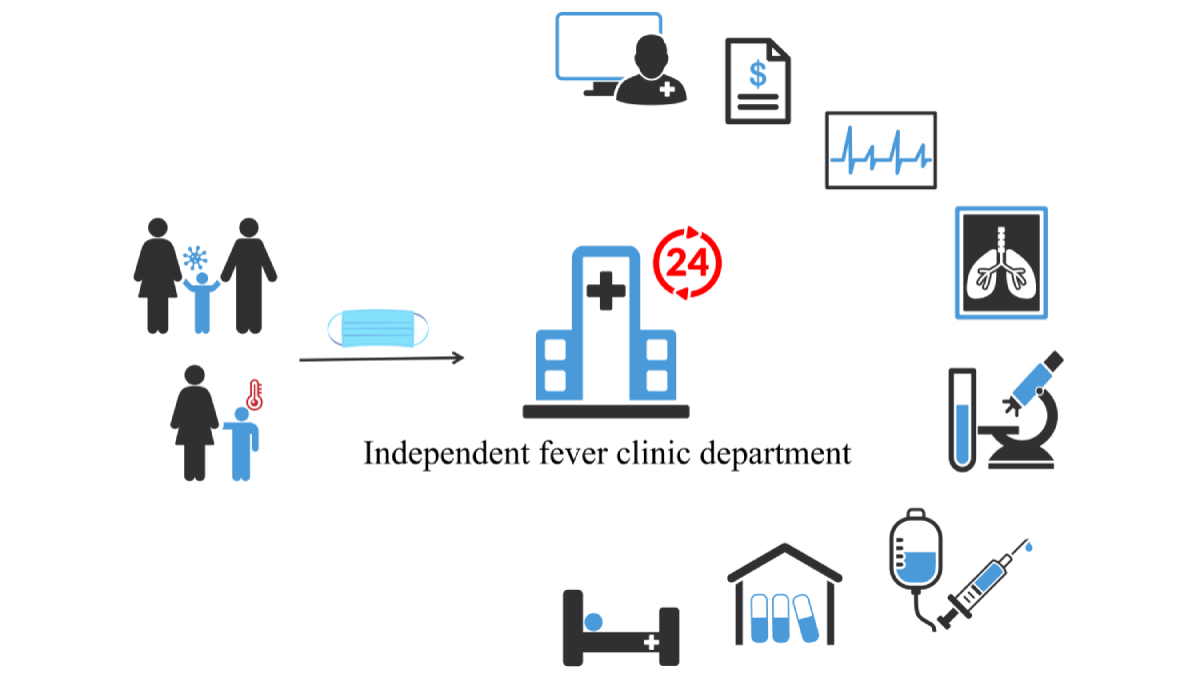
Fever clinic deployment instructions in the designated hospital.

In China, since the outbreak of COVID-19, most public hospitals have established fever clinics that can achieve individual closed-loop management in the clinics themselves (as shown in Figure 1). This enables the centralized treatment of patients with fever infections. As mandated by the National Health Commission, the allocation of resources and the operation in fever clinics must strictly adhere to established guidelines [4] to minimize the risk of hospital infection. Some hospitals even operate their fever clinics 24/7. Despite the relaxation of China’s epidemic containment policy since the end of 2022, many hospitals continue to operate their fever clinics as usual.

The presence of epidemics and the establishment of continuously operating fever clinics are altering visitation patterns, particularly among pediatric patients. Compared with adult patients, pediatric patients require more attentive care from both their guardians and medical staff, and their conditions are more prone to relapse, resulting in a continuous and intensive trend of fever clinic visits among pediatric patients during the pandemic. This poses great challenges for hospital outpatient management and the prevention of hospital-acquired infections. In this context, there is growing interest in the study of outpatient visit forecasting.

Forecasting visits to fever clinics offers numerous benefits for hospital management. Accurate and timely visit forecasts can facilitate the rational allocation of manpower and medical consumables in outpatient departments as well as the refined management and scheduling of medical equipment and facilities. Moreover, hour-level outpatient visit forecasts can provide a valuable decision-making reference for patients’ time management, enhancing efficiency for both hospitals and patients. Hourly visits to fever clinics can be characterized as a long-sequence time series in high frequency owing to their high sampling rate and large time window. The unique visitation patterns of children with fever will also certainly be reflected in the time-series data. Therefore, a peer-to-peer approach capable of uncovering the intrinsic patterns of pediatric fever clinic visit time series and establishing accurate and fine-grained visit forecast models is highly desirable.

### Related Work

Time-series analysis and forecasting have been widely applied in various fields, such as disease analysis [5-9], hospital operation management [10-14], and drug management [15-18]. Numerous studies have been conducted to forecast daily and hourly arrivals or occupancies in emergency departments. Hertzum [19] and Choudhury and Urena [20] effectively predicted hourly arrivals using autoregressive integrated moving average (ARIMA) models, whereas Becerra et al [21] studied a seasonal ARIMA (SARIMA) forecast model based on daily emergency admissions for respiratory outpatients. Cheng et al [22] and Whitt and Zhang [23] applied SARIMA with an external repressor to forecast hourly occupancy. Deep learning algorithms, such as the variational autoencoder proposed by Harrou et al [24] and the long short-term memory (LSTM) used by Etu et al [25], have also been applied to such problems, demonstrating that prediction results can significantly aid decision support in hospital management. Zhang et al [26] and Sudarshan et al [27] incorporated external variables, such as calendar and meteorological information, into the LSTM model, verifying the improvement in the accuracy of the models established in their research case.

Khaldi et al [28] investigated a hybrid model combining artificial neural networks with ensemble empirical mode decomposition, which exhibited better approximation and generalization capabilities than the benchmarking models when applied to weekly arrivals in emergency departments. Deng et al [29] proposed a hybrid ARIMA-LSTM model optimized by the backpropagation neural networks that achieved more accurate and stable predictions than the respective single models and the traditional hybrid model when forecasting weekly and monthly outpatient visits to the respiratory department. Perone [30] used single-step time-series methods and their feasible ensembles to forecast the arrival of hospitalized patients with COVID-19 presenting with mild symptoms, as well as those in intensive care units. They discovered that hybrid models were significantly better at capturing linear, nonlinear, and seasonal pandemic patterns than their respective single models on both time series.

Although numerous studies have been dedicated to forecasting outpatient visits, the time-series models used in existing hospital visit forecasting studies are limited in their ability, for they handle only a single seasonality pattern. When applied to long-sequence time series in high frequency, these models are unable to capture all seasonal patterns present in the data, resulting in the loss of data features and increased difficulty in forecasting. This is the significant drawback of single-seasonal-pattern models. Additionally, although cross-validation has been conducted in previous time-series studies to evaluate model performance, it has rarely been used as a basis for comparing the forecasting performance of models across different forecast horizons, leading to an incomplete analysis and evaluation of the predictive capabilities of different models. This study aimed to address these limitations.

### Objective

In this study, we aimed to develop a reliable forecasting model for the hospital management of fever clinics. Unlike previous studies, we focused on establishing models suitable for long-sequence time series in high frequency. The model needed to meet the following 3 requirements to facilitate our fever clinic visit forecast task. Firstly, we prioritized hourly forecasts over daily forecasts to predict fever clinic visits, as this allows for greater flexibility in hospital management and resource allocation. Second, an ideal model should be capable of making medium- to long-term forecasts (eg, 15 days) on high-frequency time-series data to aid hospital managers in making informed decisions. Finally, the model should be scalable and stable and exhibit robust performance.

To this end, we first conducted an exploratory analysis of time-series data to uncover the patterns and features of fever clinic hourly visit time series and then used an ensemble of time-series models and their combinations for fever clinic visit forecasting. For model evaluation, we used cross-validation to compare the accuracy of all models across different forecast horizons and analyzed the results. Exploratory analysis can help discover the inherent laws of data, which makes it easier to find models that fit the characteristics of the data. Cross-validation across different forecast horizons helps find models with superior scalability and stability. These are the 2 points that represent the main highlights of our research compared with existing work.

## Methods

### Ethical Considerations

The authors are accountable for all aspects of the work and are responsible for ensuring that questions related to the accuracy or integrity of any part of the work are appropriately investigated and resolved. This study was conducted in accordance with the Declaration of Helsinki (as revised in 2013). The study was approved by the Academic Ethics Committee of the Children’s Hospital of Zhejiang University School of Medicine (2020-RIB- 058). The requirement for individual consent for this retrospective analysis was waived.

### Study Participants

**Figure 2:**
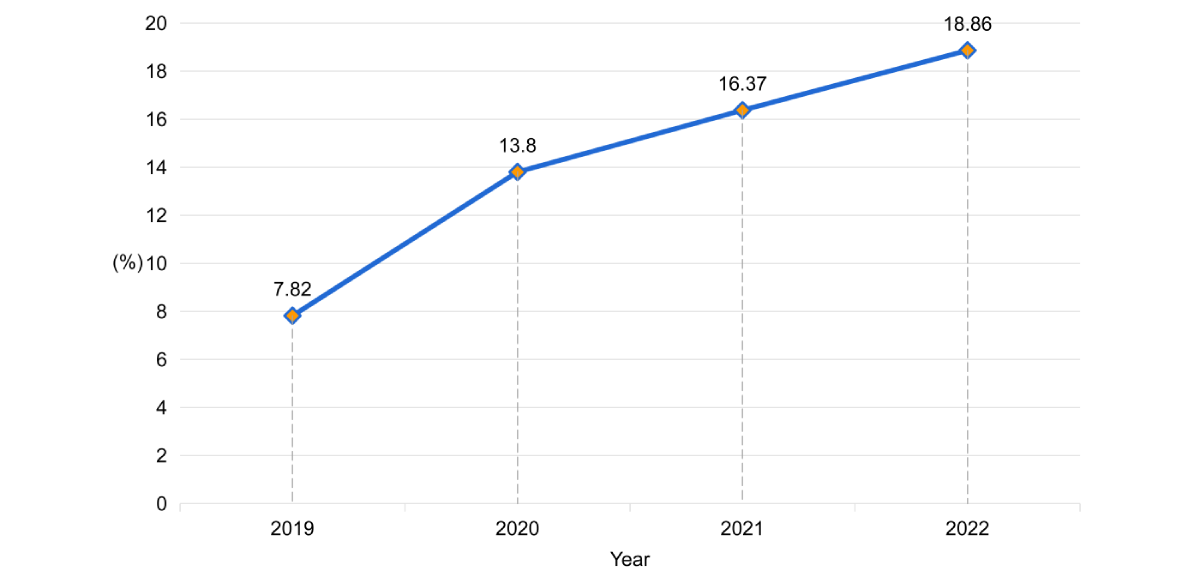
The ratio of fever clinic visits to the total number of outpatient visits over the past 4 years.

This study focused on the Children's Hospital of Zhejiang University School of Medicine, a preeminent comprehensive class A tertiary children’s hospital located in the Zhejiang province, China. In response to the COVID-19 outbreak in 2020, the hospital’s fever clinic transitioned to a 24-hour emergency operation mode, providing uninterrupted care for pediatric patients. The fever clinic operates as an autonomous department fully equipped with comprehensive medical resources. Figure 2 presents data illustrating the proportion of fever clinic visits relative to the total number of outpatient visits over the past 4 years. The data revealed a consistent increase in the proportion of fever clinic visits, with a notable surge in 2020.

### Data Collection and Preprocessing

The data set used in this study consisted of authentic data extracted from the electronic medical record (EMR) text of the aforementioned hospital’s fever clinic. Data were collected from January 23, 2020, to May 23, 2023, encompassing the onset of the COVID-19 outbreak and the phases of strict containment policy (January 2021 to November 2022) and open containment policy (December 2022 to present) implemented in the region. We tallied hourly fever clinic visits based on patients’ initial visit records in the EMR and divided each day’s 24-hour data into 24 nonoverlapping segments. Our data set comprises a time series of 29,208 data points (1217 days × 24 hours) representing hourly visits. Our data set exhibited a paucity of missing data, which we addressed by filling sporadic gaps in the hourly count with a value of 0. Outliers were identified through the remainder sequence obtained via seasonal-trend decomposition using Loess (STL) decomposition and defined as values exceeding 3 IQRs from the central 50% of the data. These outliers were subsequently smoothed using linear interpolation.

To further scrutinize our data set, we conducted statistical analyses based on children’s developmental stages as determined by their educational level [31]. The total number of patients was 1,590,909, and we divided them into the following 5 groups: infants (aged 0-2 years; n=661,425, 41.58%), preprimary children (aged 3-5 years; n=599,464, 37.68%), primary school children (aged 6-11 years; n=302,046, 18.99%), junior secondary students (aged 12-14 years; n=25,364, 1.59%), and senior secondary students (aged 15-17 years; n=2610, 0.16%). Table 1 depicts fever clinic visits for each group, revealing that the patients were predominantly infants and preprimary children.

Furthermore, we extracted International Classification of Diseases, 10th Revision disease diagnosis codes [32] from the EMR data and compiled statistics on related diseases in the fever clinic. Our results indicate that respiratory diseases constitute the largest proportion (738,635/1,590,909, 46.43%) of fever clinic cases, followed by infectious and parasitic diseases (347,199/1,590,909, 21.82%) and digestive diseases (174,814/1,590,909, 10.99%), all of which are influenced by climate change.

**Table 1:** Distribution of patients who visited the fever clinic across age groups from January 23, 2020, to May 23, 2023.

	0-2 years	3-5 years	6-11 years	12-14 years	15-17 years
Girls, n	297,590	283,567	141,768	10,887	1221
Boys, n	363,835	315,897	160,278	14,477	1389

### Exploratory Data Analysis

Before constructing forecasting models, it is imperative to comprehend the behavior of data in the time domain. Using statistical graphics and data visualization techniques, time-series patterns can be extracted and interpreted, facilitating model selection and minimizing errors.

#### Graphic Analysis

**Figure 3:**
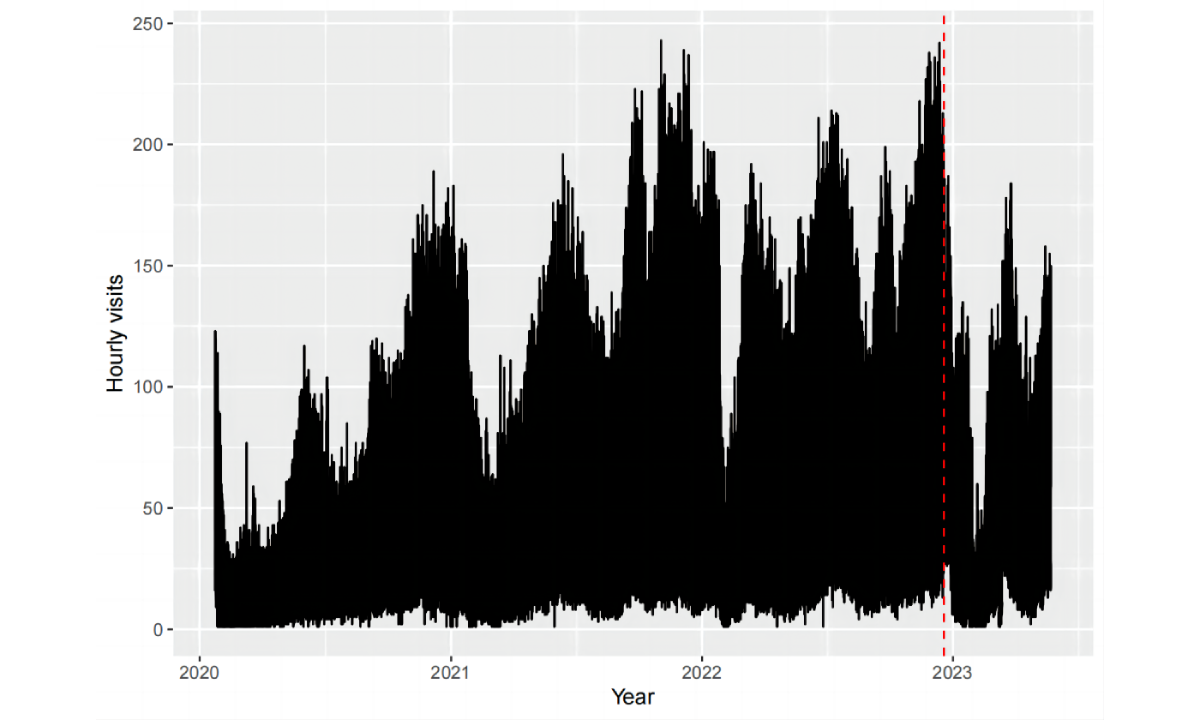
Time-series plot of hourly fever clinic visits from January 23, 2020, to May 23, 2023.

**Figure 4:**
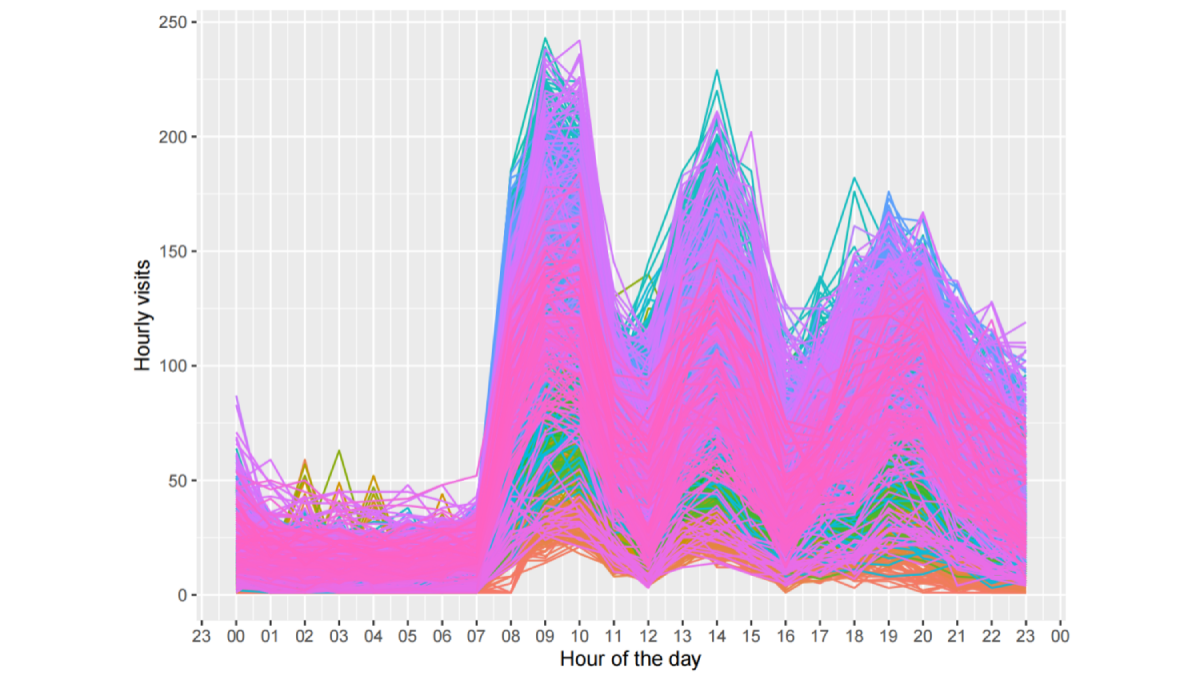
Daily seasonal patterns in the hourly fever clinic visit time series.

**Figure 5:**
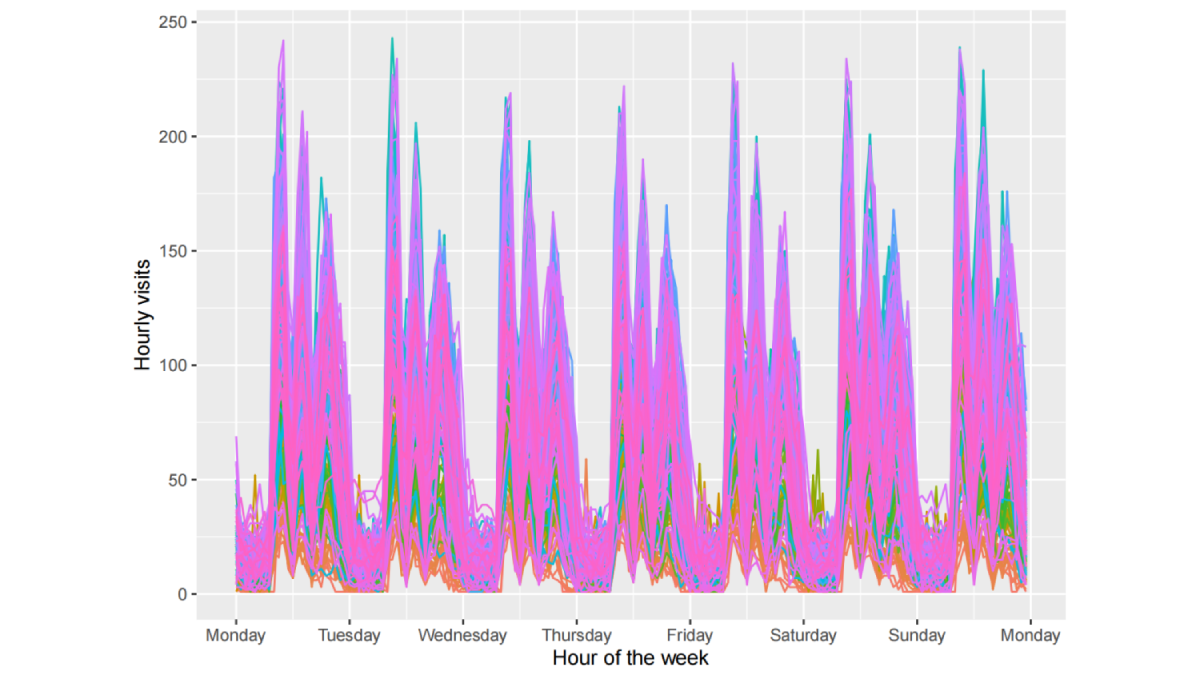
Weekly seasonal patterns in the hourly fever clinic visit time series.

**Figure 6:**
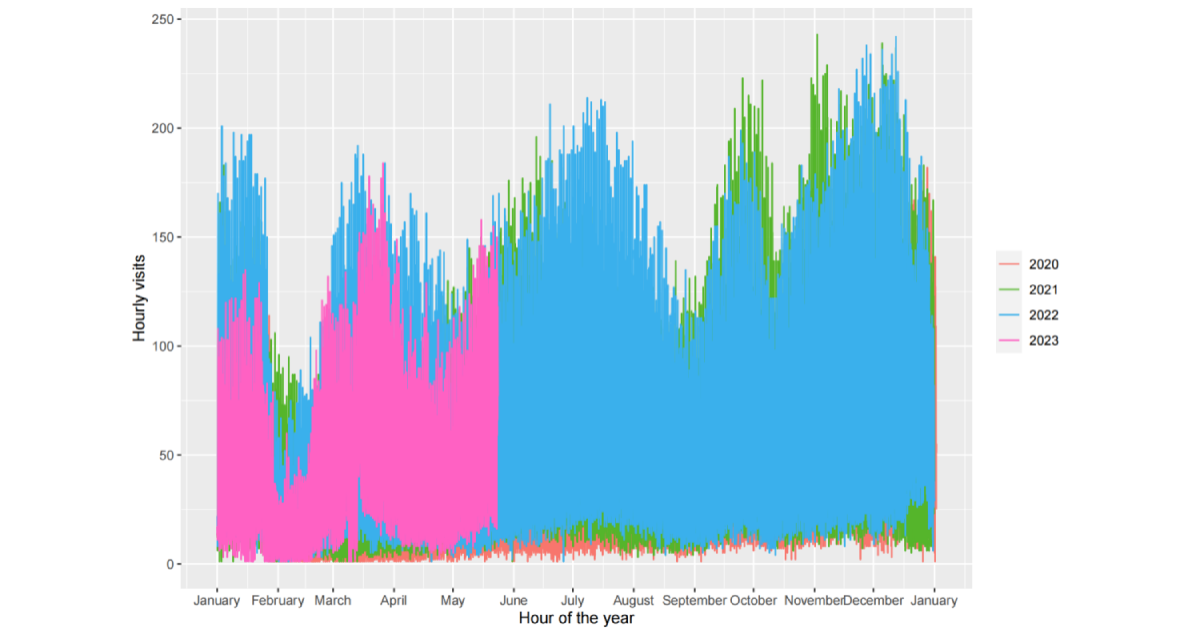
Yearly seasonal patterns in the hourly fever clinic visit time series.

Figure 3 depicts the hourly fever clinic visit time series from our data set. We plotted time-series data before and after the changes in the epidemic containment policy, with December 19, 2022, as the separation point. To identify the underlying patterns in the time series, we analyzed the data from a seasonal perspective. Given that our data are hourly, they may exhibit 3 types of seasonality: daily, weekly, and yearly. These patterns are plotted in Figures 4-6.

As illustrated in Figure 4, the diurnal pattern of fever clinic visits exhibited relatively fewer visits during the early morning hours, with 3 prominent peaks occurring at 9 AM, 2 PM, and 8 PM, indicating heightened visitation. Figure 5 reveals that visitation peaks are most pronounced on Mondays and Tuesdays, diminishing on Wednesdays and Thursdays before a resurgence from Friday through the weekend. Despite an overall decline in fever clinic visits in 2023 due to the relaxation of COVID-19 policies, the time series which consists of data spanning over 3 years still exhibits clear annual periodicity. As shown in Figure 6, values fluctuate systematically with seasonal and even monthly variations, with elevated values during winter, diminished values during spring, and peak outpatient periods coinciding with summer vacation. The location of troughs in Figure 6 appears to be influenced by movable holidays such as the Chinese New Year. From Figures 4-6, we can deduce that the time series exhibits conspicuous multiseasonal patterns, including daily, weekly, and yearly seasonalities, thereby exhibiting robust predictability.

#### Autocorrelation Function Analysis

The analysis of autocorrelation function (ACF) and partial ACF (PACF) for sample data is a crucial approach for identifying the characteristics of seasonal time series and proposing appropriate candidate models [33]. The lag *k* autocorrelation coefficient *r_k_*, as measured by ACF, quantifies the linear correlation between 2 observations, *y_t_* and *y_t−k_* can be expressed per the following formula:



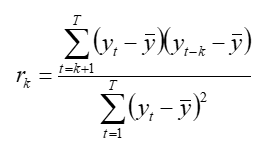



where *y_t_* represents the value of the time series at time *t,* 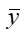 is the mean, and *T* is the length of the time series. PACF, by contrast, measures the direct correlation between *y_t_* and *y_t−k_* while isolating the effects of periods other than *k* from the analysis.

**Figure 7:**
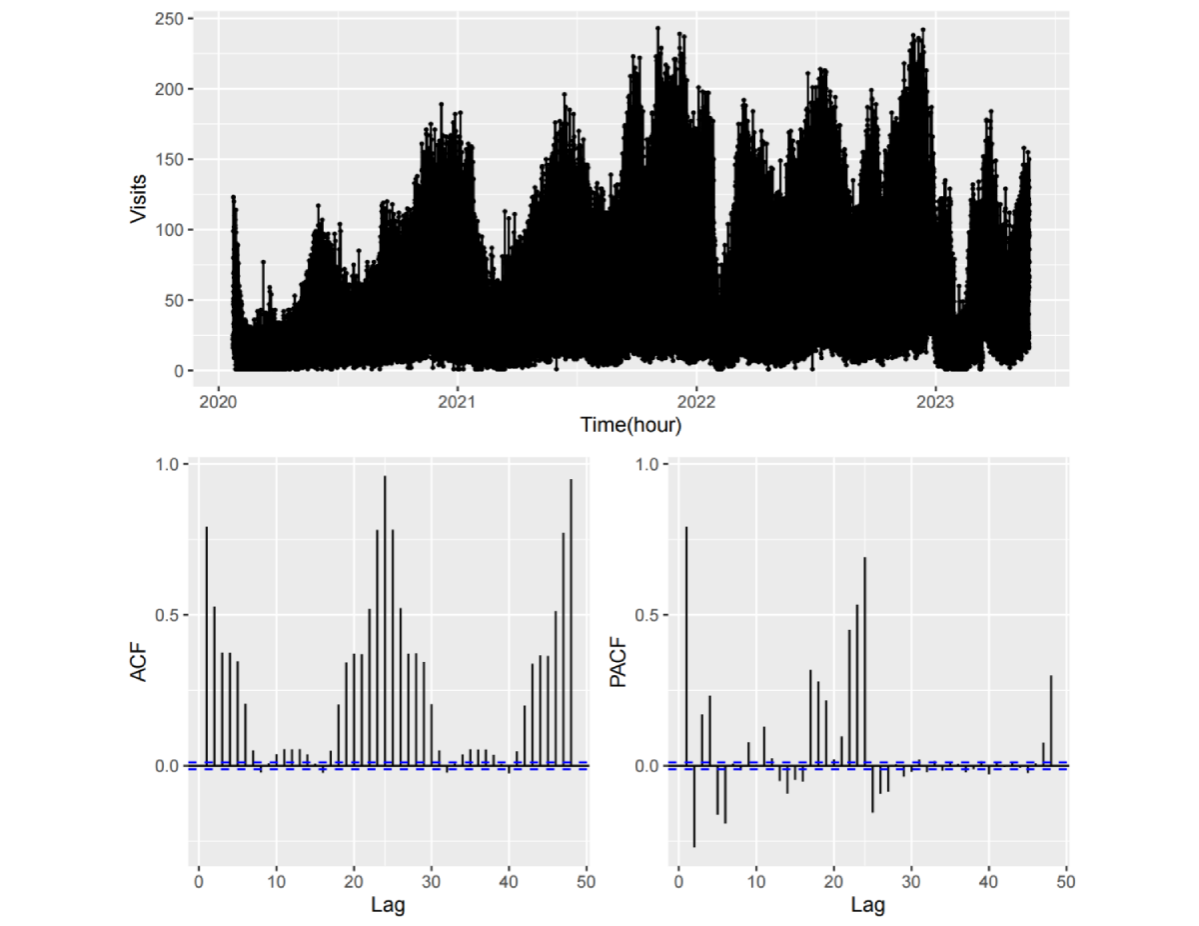
Autocorrelation function (ACF) and partial autocorrelation function (PACF) of the hourly fever clinic visit time series.

**Figure 8:**
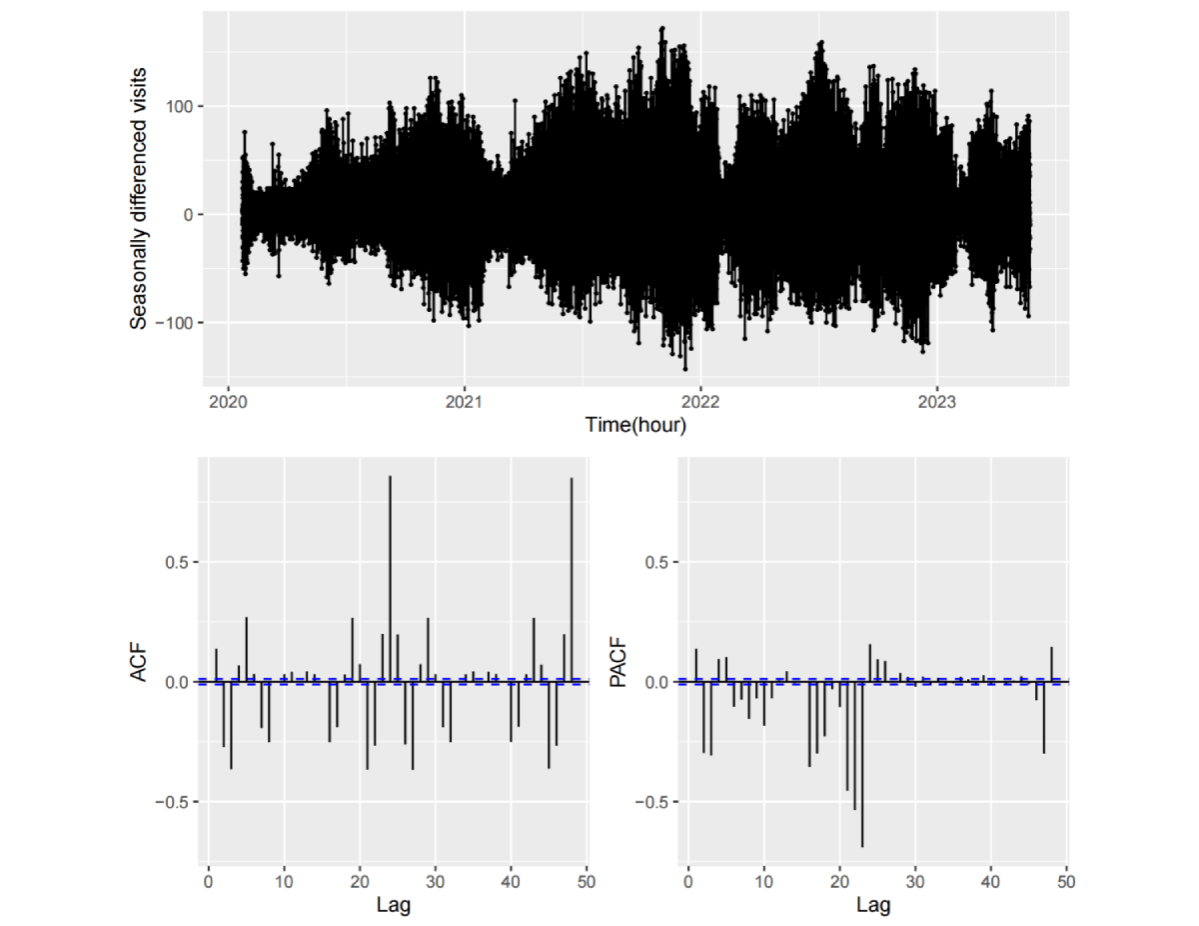
Autocorrelation function (ACF) and partial autocorrelation function (PACF) of the seasonal differential hourly fever clinic visit time series.

Figures 7 and 8 depict the sample ACF and PACF for the initial 48 lags derived from the hourly fever clinic visitation data and their corresponding seasonal differential data. As illustrated in Figure 7, the ACF values are uniformly positive and exhibit symmetrical, humped shapes with spike values occurring at multiples of 24-hour intervals, whereas the PACF values exhibit decay in seasonal lags at multiples of 24 hours. These observations suggest that the hourly visit time series is nonstationary and lacks a discernible trend, yet it exhibits strong daily seasonality. Consequently, a seasonal difference by daily period (24 hours) can be applied to generate a stationary time series. As demonstrated in Figure 8, the ACF decays exponentially to approximately 0 after 5 lags, with a downward spike at the first seasonal lag, whereas the PACF exhibits tailing off within each daily periodicity. This indicates the presence of short-term autocorrelation within the differential series as well as a strong negative autocorrelation with 1 seasonal lag. Therefore, it is feasible to achieve short-term forecasts and single seasonal (daily seasonal) forecasts through autocorrelation-based modeling.

#### STL Analysis

The decomposition of a time series can be used to assess its strength of trend and seasonality. The STL, developed by Cleveland et al [34], constitutes a filtering procedure for decomposing a time series into trend, seasonal, and remainder components in an additive manner. This methodology was subsequently extended to facilitate the decomposition of time series exhibiting multiple seasonal patterns [35].

**Figure 9:**
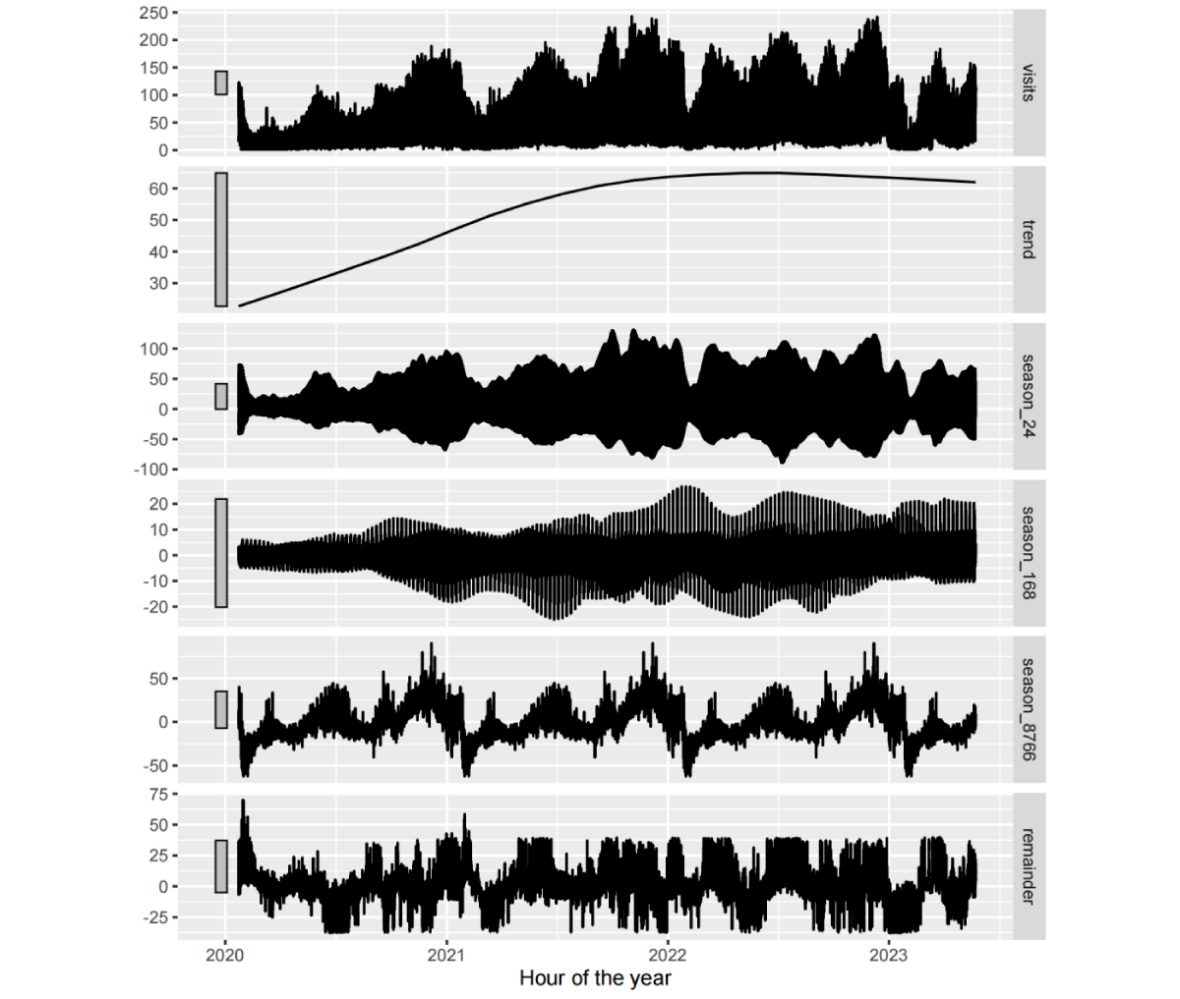
Seasonal-trend decomposition using Loess on the hourly fever clinic visit time series.

Figure 9 shows the application of STL to hourly fever clinic visit data, yielding multiple seasonal components as well as trend and remainder components. As expected, 3 seasonal patterns were evident, corresponding to the time of day (third panel), time of week (fourth panel), and time of year (fifth panel).

Note the vertical scales of all panels in Figure 9; the trend panel has the widest bar compared with the other panels, which means that the trend has the narrowest range and, consequently, accounts for only a small proportion of the variation within the data series. The weekly seasonality panel exhibits similar characteristics. In addition, the bars on the daily and yearly seasonality panels are only slightly larger than that on the data panel, indicating that the daily and yearly seasonality signals are largely related to variations in the data series.

It can be inferred that the number of visits exhibited an upward trend during the first 2 years, followed by a modest decline commencing at the end of 2022. However, the impact of this change in trend on the overall time series is negligible. Consequently, the influence of changes in the epidemic containment policy on the data can be ignored. Concurrently, the weekly seasonality inherent within the series is relatively weak and exerts minimal influence on temporal variations within the time series, whereas daily and yearly seasonalities exert a more pronounced effect. Furthermore, it is evident that the daily seasonal pattern undergoes temporal variations, whereas the yearly seasonal pattern appears relatively fixed. Therefore, for long-term forecasting, it is advisable to incorporate yearly seasonal lag data. Finally, the panel at the bottom exhibits large random fluctuations owing to its slightly larger size relative to the data panel. This suggests the presence of additional nonlinear signals infiltrating the residual components, potentially attributable to outliers or unaccountable factors.

### Theory and Calculations

This section delineates the models used in this study and explains the forecast accuracy measures used in the evaluation of model performance.

#### Forecasting Models

Before conducting experiments, we established a benchmark method as the standard to ensure that the performance of the other selected models surpasses that of it. As a benchmark approach, it is reasonable to consider the prevailing seasonality in the time series. We set the seasonal naive (SNaive) method as our benchmark, given its utility for highly seasonal data. This method sets each forecast such that it is equal to the last observed value from the corresponding season. Here, the predicted value for the forecast horizon *h* is considered to be equivalent to the observed value from the previous day. Formally, the forecast for time *T*+*h* is expressed as



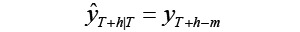



where *m* represents the specified seasonal period. This approach requires no parameterization or setup and is frequently used as a benchmark method rather than a model of choice.

Time series exhibiting trends or seasonality are not stationary, as their statistical properties vary over time. In such instances, nonstationary time series can be rendered stationary through the application of differencing techniques. The ARIMA model constitutes a combination of differencing with autoregressive and moving average (MA) components, which were first proposed by Box and Jenkins [36], and is commonly denoted as ARIMA(p,d,q). Autoregressive refers to the regression of a variable in the model on its own lagged or prior values, whereas MA incorporates the dependency between an observation and a residual error derived from an MA model applied to lagged observations. However, the ARIMA model is only applicable to nonseasonal data. The SARIMA model, denoted as ARIMA(p,d,q)(P,D,Q)_m_, was derived by incorporating additional seasonal terms into the ARIMA model. It should be noted that the SARIMA model can only specify a single seasonal period parameter, rendering it capable of handling only single seasonality. A mathematical exposition of the ARIMA and SARIMA models can be found in Multimedia Appendix 1.

As demonstrated in the *STL analysis* section, STL has been applied to the hourly fever clinic visit time series exhibiting multiple seasonality. This can be conceptualized as decomposition into 3 seasonal components and seasonally adjusted components. The STL method is based on the performance of weighted local regressions (Loess) on seasonal indices and the trend, with Loess constituting a methodology for estimating nonlinear relationships. This approach confers benefits upon statistical methods by providing a more versatile and robust decomposition procedure than their intrinsic mechanism [37]. The improved algorithm for multiple seasonality uses an iterative method to obtain seasonal components sequentially in ascending order of cycles and finally to compute the trend component in the last iteration [35]. The forecasting approach based on the STL method is defined as the STL forecasting (STLF) model. In this approach, each seasonal component is forecasted by the SNaive method corresponding to the seasonal lags, whereas seasonally adjusted data are forecasted using a nonseasonal ARIMA model. Evidently, this forecasting approach has an advantage in handling multiple seasonal patterns for a long time series, in contrast to the SARIMA model, which is limited to handling single seasonality.

Artificial neural network is based on a structure comprising an input layer, hidden layers, and an output layer, facilitating the modeling of complex nonlinear relationships between response variables and their predictors. The neural network autoregressive (NNAR) model uses lagged values of the time series as inputs to the neural network, with the last observed values from the corresponding season also incorporated as inputs. Generally, the notation NNAR(p,P,K)_n_ is used to denote the presence of *p* lag inputs, *P* seasonal lag inputs, and *K* neuron nodes in the hidden layer, where *m* represents the seasonal period. A model can be defined as *y_t_=f(y_t__−1_,y_t−2_,...,y_t−p_,y_t−m_,...y_t−Pm_)+ ε_t_*, where f represents the nonlinear function of the feed-forward network with a single hidden layer, and *ε_t_* is the residual series. In contrast to traditional time-series methods, the network may be applied iteratively, with predictions incorporated as inputs alongside historical data when forecasting additional steps ahead.

In addition, hybrid combinations of the aforementioned models include hybrid SARIMA-STLF, hybrid NNAR-STLF, hybrid SARIMA-NNAR, and hybrid SARIMA-NNAR-STLF. In these hybrid models, multiple forecasts are combined by averaging the forecasts of individual models. This constitutes a straightforward yet effective means of enhancing the forecast accuracy [38]. Moreover, fixed weights facilitate the identification of optimal solutions more effectively than weights that are based on the random statistical variables derived from changing data.

#### Accuracy Measures

We used root mean squared error (RMSE) and mean absolute error (MAE) to assess the performance of the applied models. Given that the training set is denoted as {*y_1_,y_2_,...y_T_*} and the testing set as {*y_T+1_,y_T+2_,...*}, the forecast deviation between the actual observations *y_t_* on the testing set and the corresponding forecasts 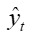 can be denoted as 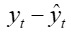. The formulas for calculating each of these metrics are as follows:



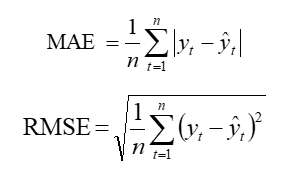



where *n* represents the total number of observations for the evaluation process. Both RMSE and MAE are scale-dependent metrics. RMSE operates on the principle of averaging errors and is more sensitive to outliers, whereas MAE is calculated from the median of errors and is more robust to outliers.

## Results

### Overview

In this section, we present the results of the evaluations of all the candidate forecasting models. Hourly visits to the fever clinic were forecasted for the subsequent 15 days, with accuracy calculated based on the forecasted values over this period. The 8 evaluated models were SNaive (as a benchmark), SARIMA, STLF, NNAR, and combinations of these models (excluding SNaive), hybrid SARIMA-STLF, hybrid NNAR-STLF, hybrid SARIMA-NNAR, and hybrid SARIMA-NNAR-STLF. For model training, we used data from January 23, 2020, to April 23, 2023, as the training set and data from April 24, 2023, to May 8, 2023, as the testing set. The performances of the models were compared based on the accuracy of their forecasting results on the testing set.

### Model Estimation

Given that the SNaive, SARIMA, and NNAR models are only capable of handling a single seasonal period, we used a daily season period as the seasonal frequency parameter for hourly data. This corresponds to a lag of 24 in SNaive and m=24 in both SARIMA and NNAR. The specifications and estimations for all the models are detailed in Tables 2 and 3. For the selection of hyperparameters and fitting of model parameters, we used the fable toolkit in the R programming language (R Core Team) for implementation.

The SARIMA modeling process was implemented using a variation of the Hyndman-Khandakar algorithm [39], in which parameter D was chosen by an extended Canova-Hansen test [40], and d was chosen through successive Kwiatkowski-Phillips-Schmidt-Shin unit-root tests [41]. Once d and D were determined, a stepwise search was conducted to traverse the ARIMA order space from the initial candidate parameters, selecting values for p, q, P, and Q by minimizing the corrected Akaike’s information criterion until the residuals met the white noise conditions. The modeling process for nonseasonal ARIMA was similar, with the exception that the seasonal hyperparameters were set to 0 (P=D=Q=0). After identifying the model orders (hyperparameters p, d, q, P, D, and Q) with the lowest corrected Akaike’s information criterion, the model parameters were estimated on the training set using the maximum log-likelihood estimation. In this study, the best-fitting models for SARIMA and STLF on our training data were ARIMA(0,0,5)(0,1,1)_24_ for SARIMA and ARIMA(2,1,2) for STLF. The estimated coefficients for these models are listed in Table 4.

For the NNAR model, the seasonal parameter P was set to 1, and the p parameter was selected from the optimal linear autoregressive(p) model fitted to the seasonally adjusted data (obtained through STL) according to the AIC. The k parameter was rounded to the nearest integer of (p+P+1)/2. In this study, the best-fitting model for this approach was an average of 20 networks NNAR(44,1,22)_24_, each consisting of a 44×22×1 network with inputs {*y_t−1_,y_t−2_,...y_t−44_*} and 22 neurons in the hidden layer. For 1-step-ahead (1 hour ahead) forecasting, available historical inputs were used, whereas for 2-step-ahead forecasting, the 1-step forecast was used as an input along with historical data. This process was executed iteratively until all the required forecasts were computed [38].

**Table 2:** Summary of the specifications and estimations for different models.

Model and specification		σ^2^
**SNaive^a^**		
	SNaive(24)	156.9503
**SARIMA^b^**		
	ARIMA^c^(0,0,5)(0,1,1) [24]	103
**NNAR^d^**		
	NNAR(44,1,22) [24]	87.6
**STLF^e^**		
	ARIMA(2,1,2)	55.04
	SNaive(24)	1.4266
	SNaive(168)	0.2336
	SNaive(8766)	0.0045

^a^SNaive: seasonal naive.

^b^SARIMA: seasonal autoregressive integrated moving average.

^c^ARIMA: autoregressive integrated moving average.

^d^NNAR: neural network autoregressive.

^e^STLF: seasonal and trend decomposition using Loess forecasting.

**Table 3:** Summary of information criterion for autoregressive integrated moving average (ARIMA) models.

Information criterion	ARIMA(0,0,5)(0,1,1) [24]	ARIMA(2,1,2)
AIC^a^	212,731.5	195,029
AICc^b^	212,731.5	195,029
BIC^c^	212,789.3	195,070.2
Log likelihood	–106,358.8	–97,509.48

^a^AIC: Akaike information criterion

^b^AICc: corrected Akaike information criterion.

^c^BIC: Bayesian information criterion.

**Table 4:** Estimated coefficients of autoregressive integrated moving average (ARIMA) models.

Model and term			Coefficient (SE)		*t*-statistic		*P* value
**ARIMA(0,0,5)(0,1,1) [24]**							
	MA^a^(1)	0.3226 (0.0061)		52.49		<.001	
	MA(2)	0.2177 (0.0063)		34.61		<.001	
	MA(3)	0.1728 (0.0057)		30.21		<.001	
	MA(4)	0.1189 (0.0060)		19.78		<.001	
	MA(5)	0.1088 (0.0058)		18.82		<.001	
	SMA^b^(1)	–0.6929 (0.0046)		–150		<.001	
**ARIMA(2,1,2)**							
	AR^c^(1)	0.6138 (0.0655)		9.37		<.001	
	AR(2)	–0.0276 (0.0131)		–2.11		.08	
	MA(1)	–1.4031 (0.0652)		–21.51		<.001	
	MA(2)	0.4333 (0.0612)		7.08		.01	

^a^MA: moving average.

^b^SMA: seasonal moving average.

^c^AR: autoregressive.

### Forecasting Results

**Figure 10:**
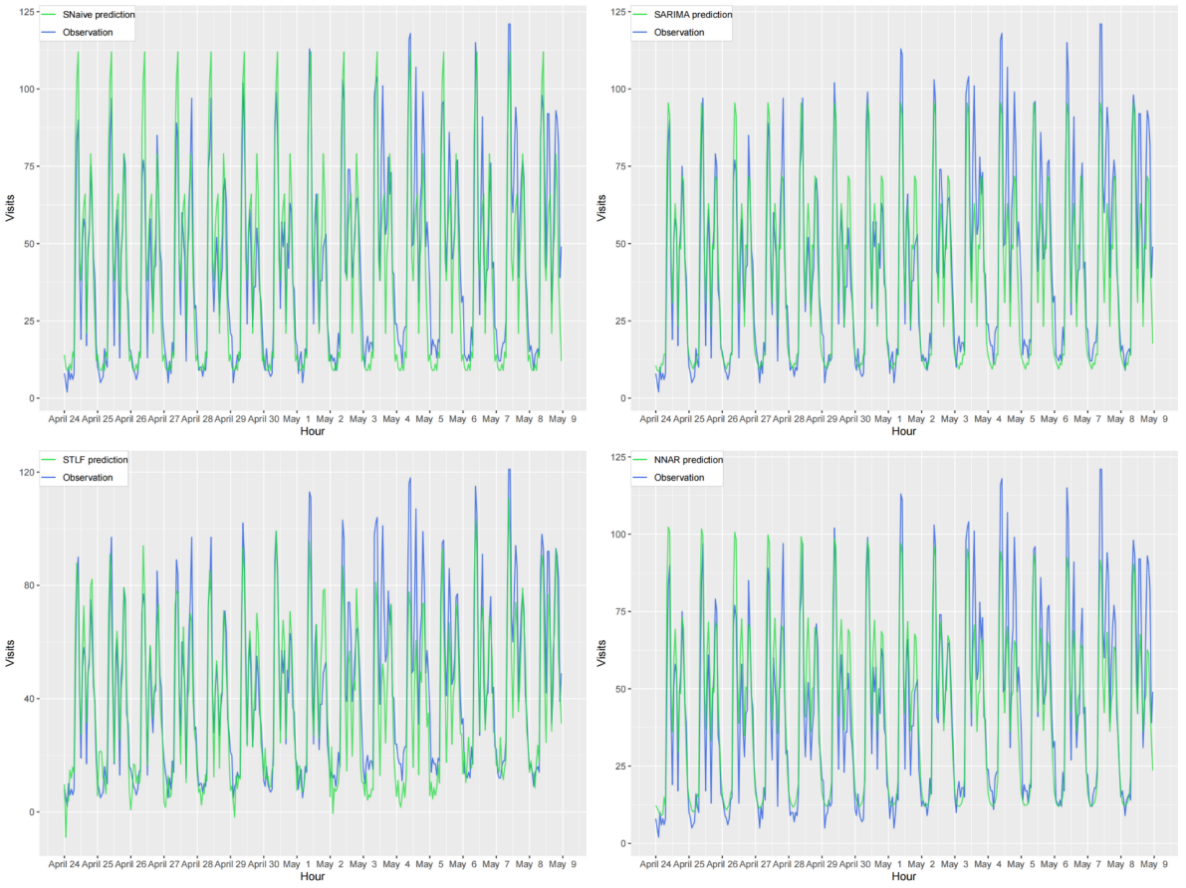
Comparison of 15-day forecasts generated using single models with actual observations. NNAR: neural network autoregressive; SARIMA: seasonal autoregressive integrated moving average; SNaive: seasonal naive; STLF: seasonal and trend decomposition using Loess forecasting.

**Figure 11:**
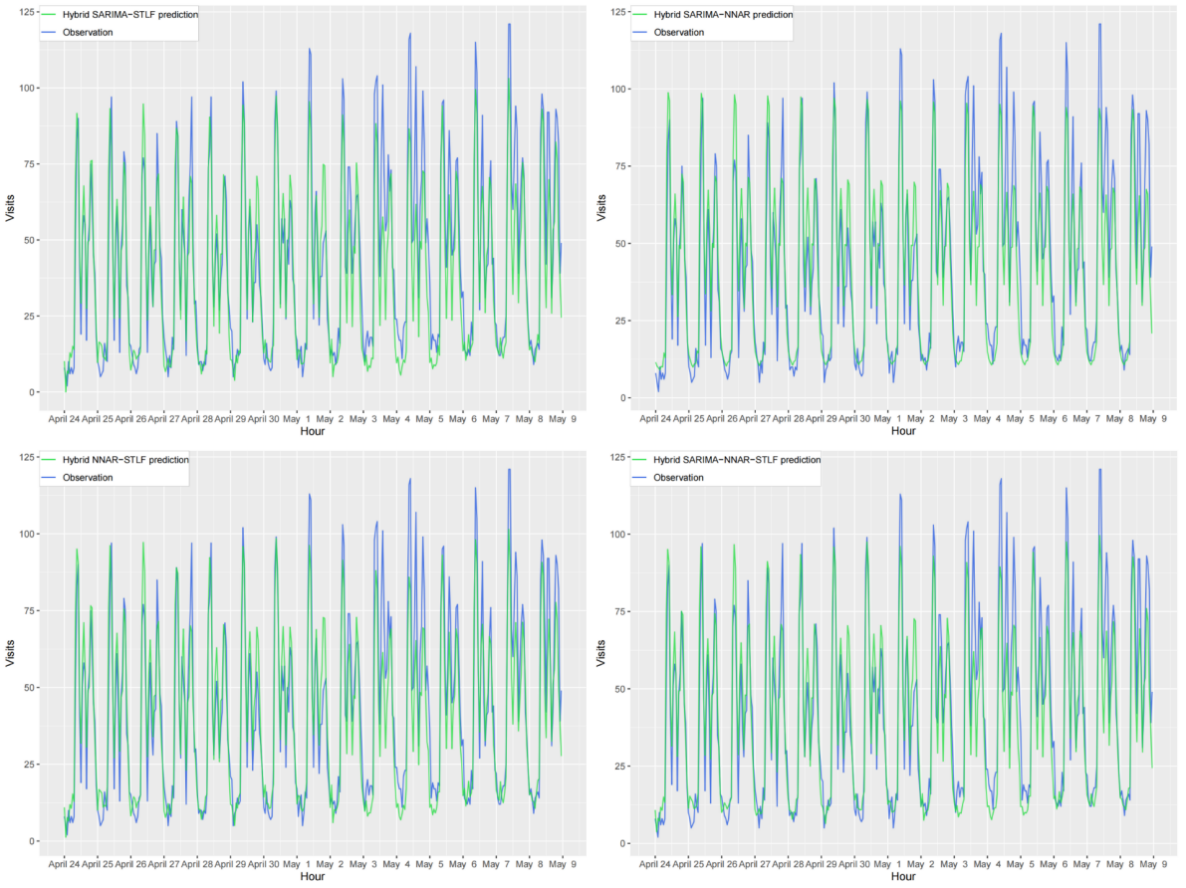
Comparison of 15-day forecasts generated using hybrid models with actual observations. NNAR: neural network autoregressive; SARIMA: seasonal autoregressive integrated moving average; STLF: seasonal and trend decomposition using Loess forecasting.

The forecasting results of all the evaluated models are shown in Figures 10 and 11. The distribution of the predicted values varies among the models. However, for short-term future data, each model appears to be capable of capturing the majority of their hourly features. It is evident that the prediction and observation lines of all the models are in reasonable agreement. However, as the forecast horizon increases, the agreement between the predicted and actual values of each model diminishes significantly. For further quantitative comparison of the model performance, we calculated the forecast errors within the subsequent 15 days for each model using the accuracy metrics RMSE and MAE. All results are presented in Table 5, which reveals that the hybrid SARIMA-NNAR-STLF model has both the lowest MAE (8.16) and the lowest RMSE (11.09), whereas STLF performs the worst.

**Table 5:** Accuracy metrics for all the models on forecasts for the subsequent 15 days (April 24, 2023, to May 8, 2023).

Model	RMSE^a^	MAE^b^
Hybrid NNAR^c^-STLF^d^	11.22	8.38
Hybrid SARIMA^e^-STLF	11.67	8.54
Hybrid SARIMA-NNAR	11.24	8.18
Hybrid SARIMA-NNAR-STLF	11.09	8.16
NNAR	11.65	8.58
SARIMA	11.51	8.33
STLF	13.03	9.69
SNaive^f^	13.02	9.66

^a^RMSE: root mean squared error.

^b^MAE: mean absolute error.

^c^NNAR: neural network autoregressive.

^d^STLF: seasonal and trend decomposition using Loess forecasting.

^e^SARIMA: seasonal autoregressive integrated moving average.

^f^SNaive: seasonal naive.

### Cross-Validation

**Figure 12:**
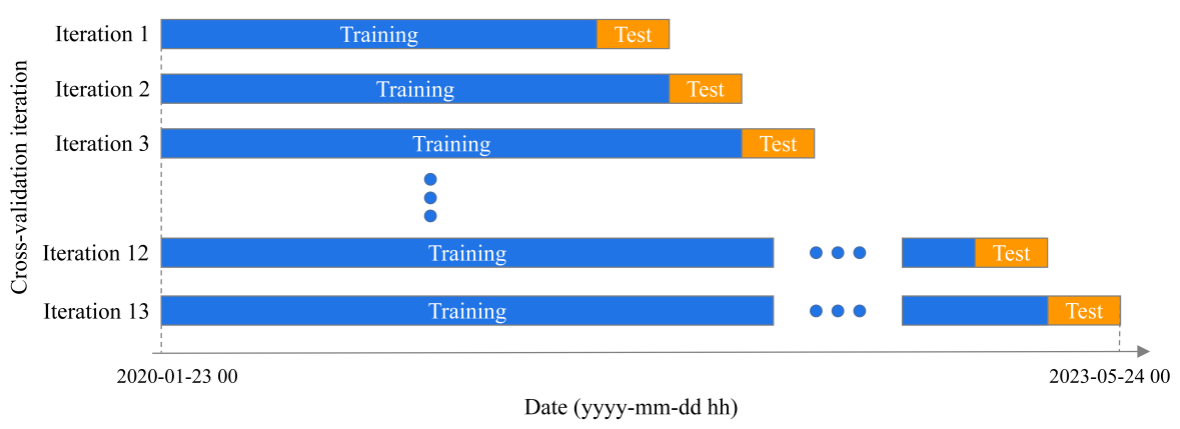
Illustration of the rolling forecasting origin cross-validation methodology.

To mitigate the risk of overfitting and determine the optimal hyperparameters for our models, we employed a cross-validation technique based on a rolling forecasting origin [42]. The forecasting origin was advanced incrementally by a fixed number of observations, with forecasts generated at each origin. As the origin progressed, the testing set was incorporated into the training set for subsequent iterations. Figure 12 illustrates the construction of our cross-validated training and testing sets. To ensure the complete coverage of each month in the testing set, we initiated our analysis with a training set comprising 19,848 observations (827 days), incrementing the size of successive training sets by 720 steps (30 days) with each iteration. This allowed us to generate 1-to-720-step-ahead forecasts and conduct 13 iterations of cross-validation to evaluate the forecasts throughout the entire year. During these iterations, the largest validation set spanned from January 23, 2020, to May 23, 2023, with the training set ranging from January 23, 2020, to April 23, 2023, and the testing set from April 24, 2023, to May 23, 2023.

Forecast accuracy was computed by averaging over the testing sets. This cross-validation method is well suited to account for the temporal dependency between observations in time series, effectively mitigating overfitting while providing a more robust evaluation of model performance [43]. Furthermore, the construction of a rolling length training set is advantageous for validation in multiseasonality cases. The forecast accuracy of all the models for the subsequent 15 days, as determined by cross-validation, is presented in Table 6. The results indicate that the hybrid NNAR-STLF model is the optimal model for this case, as it has both the lowest RMSE (20.1) and the lowest MAE (14.3).

**Table 6:** Accuracy metrics of all the models for the 15-day forecast by cross-validation.

Model	RMSE^a^	MAE^b^
Hybrid NNAR^c^-STLF^d^	20.1	14.3
Hybrid SARIMA^e^-STLF	21.1	14.6
Hybrid SARIMA-NNAR	22.7	15.5
Hybrid SARIMA-NNAR-STLF	20.4	14.5
NNAR	22.2	15.5
SARIMA	24.1	16.3
STLF	20.8	15.5
SNaive^f^	25.3	17.7

^a^RMSE: root mean squared error.

^b^MAE: mean absolute error.

^c^NNAR: neural network autoregressive.

^d^STLF: seasonal and trend decomposition using Loess forecasting.

^e^SARIMA: seasonal autoregressive integrated moving average.

^f^SNaive: seasonal naive.

### Forecast Horizon Accuracy

**Figure 13:**
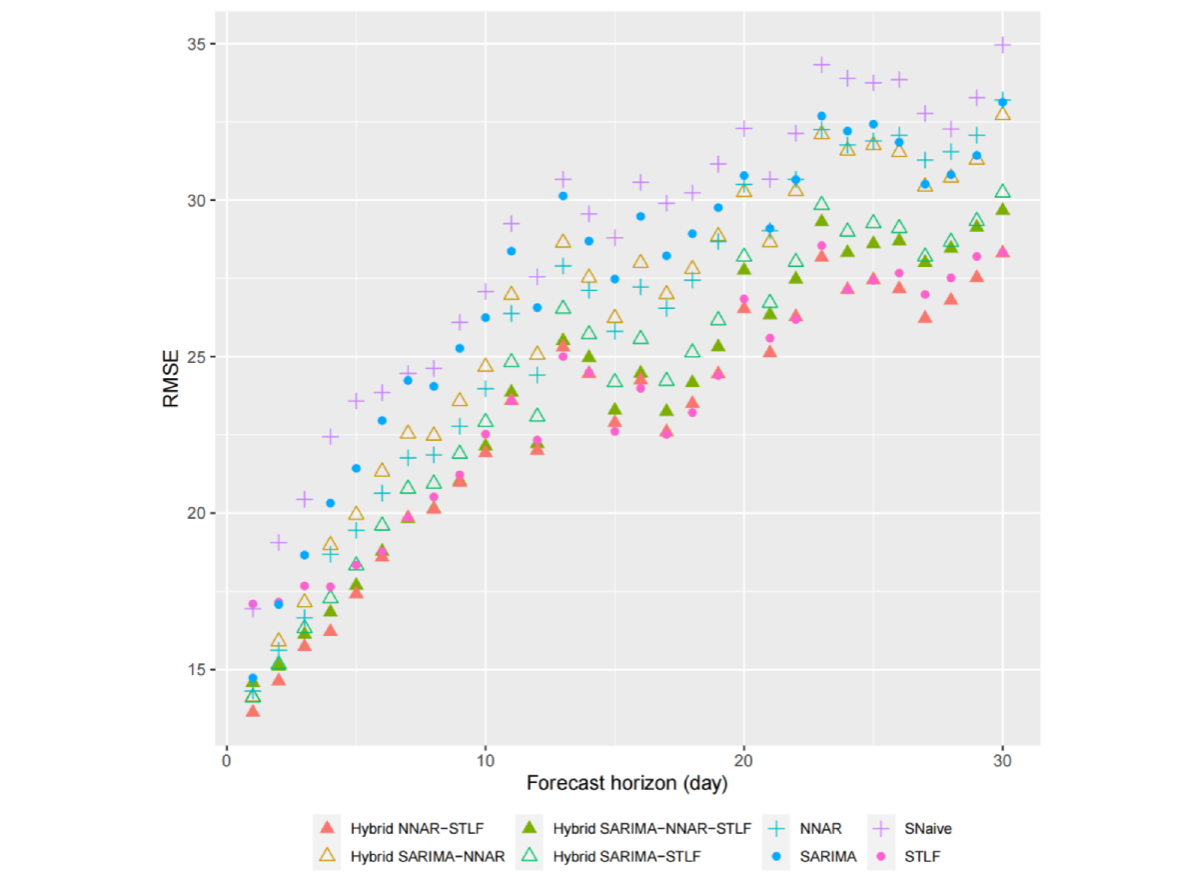
Root mean squared error (RMSE) values calculated for different forecast horizons ranging from 1 to 30 days ahead. NNAR: neural network autoregressive; SARIMA: seasonal autoregressive integrated moving average; SNaive: seasonal naive; STLF: seasonal and trend decomposition using Loess forecasting.

**Figure 14:**
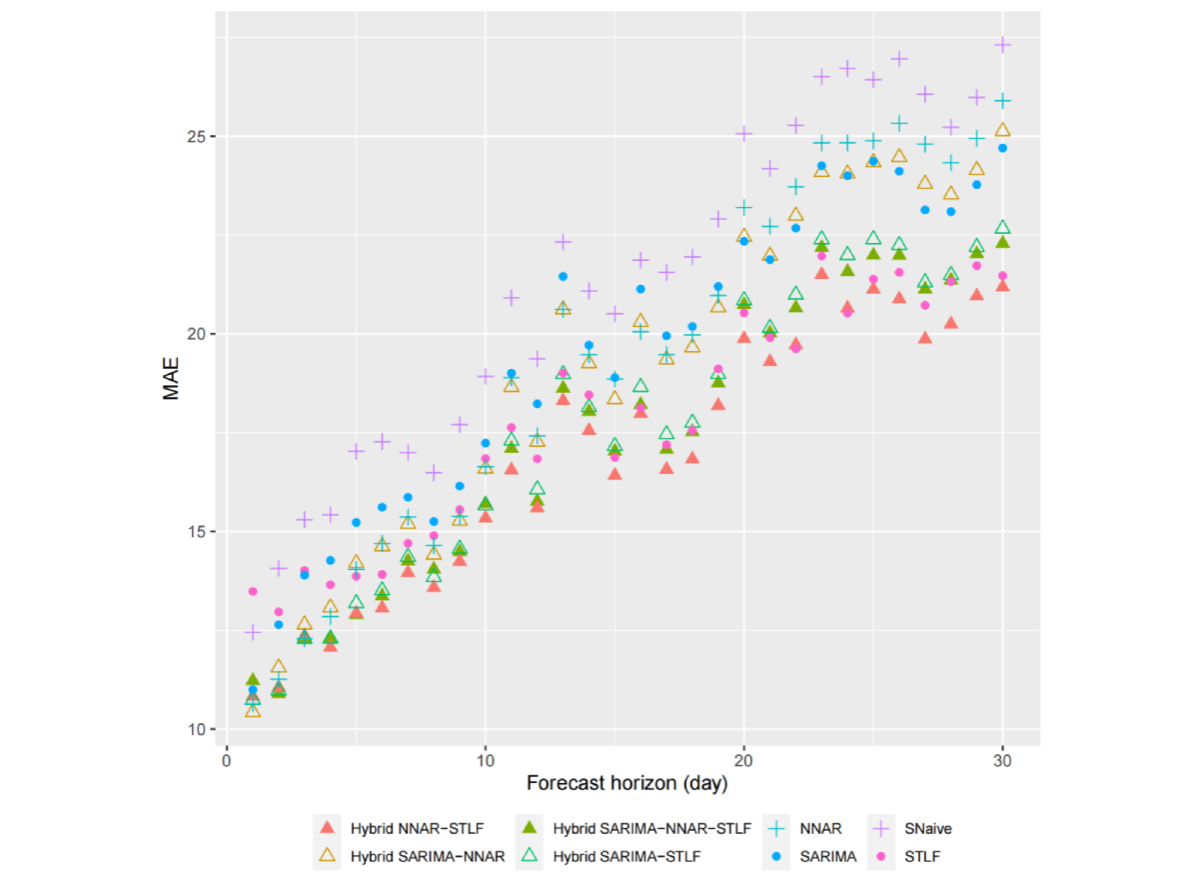
Mean absolute error (MAE) values calculated for different forecast horizons ranging from 1 to 30 days ahead. NNAR: neural network autoregressive; SARIMA: seasonal autoregressive integrated moving average; SNaive: seasonal naive; STLF: seasonal and trend decomposition using Loess forecasting.

Although cross-validation has been used in previous studies for performance evaluation, it has rarely been conducted across different forecast horizons. In this study, we not only assessed the accuracy of hourly forecasts for fever clinic visits within a 15-day window but also examined the accuracy of various forecast horizons to provide a more robust basis for identifying the optimal model. Forecast errors across forecast horizons ranging from 1 to 30 days ahead were compared using cross-validation. Specifically, the forecast horizon accuracy was calculated by comparing all the predicted values with their corresponding observed values in the hourly series within a specific 1-day range, representing the model’s predictive power on the i-th day ahead (i=1,...,30). As such, the accuracy metrics for different forecast horizons are independent of one another. The results of our analysis are presented in Figures 13 and 14, which depict the RMSE and MAE values for all the models across different forecast horizons. The calculation of RMSE and MAE was based on the average value obtained across all rolling testing sets in cross-validation.

**Figure 15:**
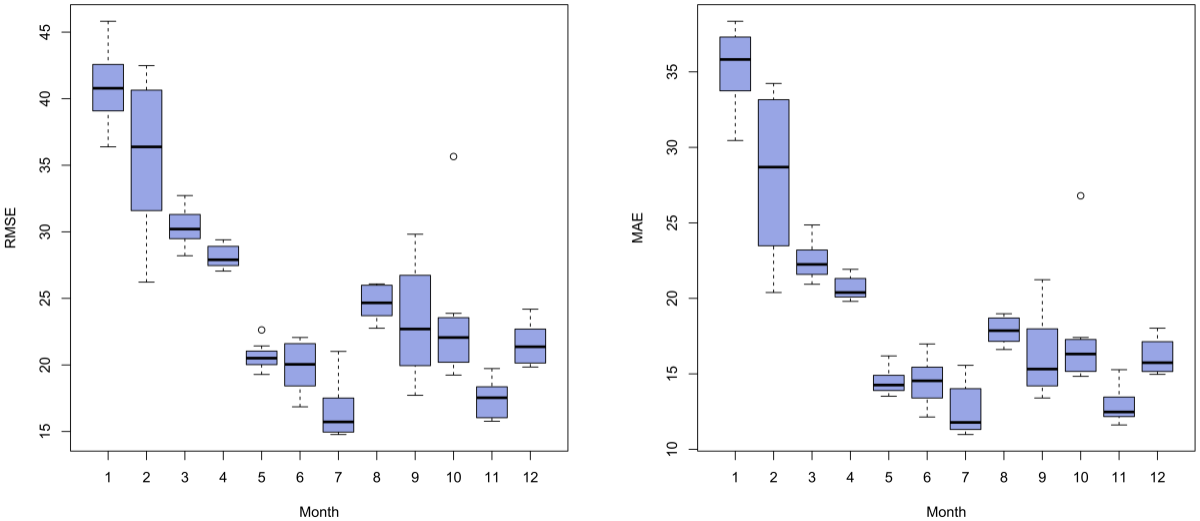
Monthly aggregated root mean squared error (RMSE) and mean absolute error (MAE) box plot.

As shown in Figures 13 and 15, for all the models, the RMSE and MAE values exhibit an overall upward trend as the forecast horizon increases, although not strictly monotonically in some cases. In general, the further ahead we forecast, the greater the uncertainty associated with our predictions. Moreover, the disparity in forecast accuracy among the models also increases with the number of days ahead of the forecast, indicating a widening gap in performance.

Among single models, STLF exhibited superior predictive performance only when forecasting more than 5 days in advance, with the RMSE and MAE values remaining relatively low, especially when forecasting more than 10 days in advance. This highlights the model’s advantages in medium- to long-term forecasting. Compared with the benchmark SNaive model, the largest difference in RMSE occurred at a forecast lead time of 17 days, with STLF achieving a value of 22.5 (7.4 lower than that of SNaive), whereas the largest difference in MAE occurred at a forecast lead time of 24 days, with STLF achieving a value of 20.5 (6.2 lower than that of SNaive). In addition, the NNAR model exhibited considerable advantages in short-term forecasting, particularly for forecasts that were 1 to 3 days in advance, with both RMSE and MAE values being the lowest among all the single models (RMSE: 14.3, 15.6, and 16.7; MAE: 10.6, 11.3, and 12.3). However, its performance in medium- to long-term forecasting is more modest.

For hybrid models, the performance was generally superior to that of their constituent single models. Comparing the results for both single and hybrid models, the hybrid NNAR-STLF model exhibited the lowest values for both RMSE and MAE across nearly all forecast horizons, with RMSE ranging from 13.6 (1st day ahead) to 28.3 (30th day ahead) and MAE ranging from 10.8 (1st day ahead) to 21.5 (23rd day ahead). It is evident that the hybrid NNAR-STLF model outperforms in all 3 cases (short-, medium-, and long-term forecasting), indicating that it may be considered the optimal model. However, not all the hybrid models performed well. For example, the hybrid SARIMA-NNAR model exhibited a relatively poor performance compared with both single and other hybrid models. Nonetheless, on the whole, the hybrid approach did have a positive impact on forecasting.

## Discussion

### Principal Findings

The hourly visit data from our hospital’s fever clinic can be characterized as a time series of long sequences in high frequency. Through exploratory analysis using data visualization, ACF, and STL methods, we observed that the time series exhibited multiseasonality and nonlinearity in its temporal patterns. To achieve our goal of generating hourly forecasts of fever clinic visits in the medium- to long-term horizons, we evaluated an ensemble of individual and hybrid models. In selecting and combining models, we considered their ability to capture multiseasonality and handle nonlinear features. Our analysis revealed that hybrid models generally outperformed individual models, with the hybrid NNAR-STLF model emerging as the optimal model for our purposes. It exhibited the smallest error in the 15-day forecast horizon (RMSE at 20.1 and MAE at 14.3) and demonstrated a stable advantage in prediction accuracy across forecast horizons ranging from 1 to 30 days ahead. This indicates that it possesses strong scalability and generalization capabilities for predicting multiseasonal periods in time-series data.

Hourly forecasts of fever clinic visits can be leveraged to enhance intelligent outpatient management and provide a sound basis for resource allocation at multiple levels. On the one hand, hospitals can use forecast results to implement flexible scheduling strategies, such as adjusting the number of doctors on duty and modifying registration limitations for specialist doctors and their working hours. On the other hand, during peak seasons and times, hospitals can adapt their facilities and human resources, including the number of service windows, medical technicians, nurses, and outpatient volunteers, to better meet the demands of outpatient operations in accordance with the forecast results. Furthermore, accurate visit forecasts can be used to schedule patient appointments and recommend optimal visitation times, thereby improving efficiency for both hospitals and patients. It is evident that hourly visit forecasts can more effectively support these requirements.

### Interpretation of the Findings

#### Single Models

SNaive and SARIMA are applicable only to time series with a single seasonal pattern. However, the time series for hourly fever clinic visits exhibits 3 seasonal patterns: daily, weekly, and yearly. This results in poor forecast accuracy for both SNaive and SARIMA. By contrast, NNAR takes lagged values as input to the neural network, establishing a more complex nonlinear relationship between forecasts and historical observations than statistical models, allowing it to capture the asymmetry of cycles. However, as it uses forecast values from previous steps as variables for subsequent steps, errors can propagate through the forecast process. In addition, its use of only a single seasonal lag as a forecast variable limits its effectiveness in medium- to long-term forecasting. STLF uses a strategy that captures more comprehensive data characteristics during the forecasting process. It first uses the SNaive method to forecast 3 seasonal components separately and then applies the ARIMA model to forecast seasonally adjusted data. Because hourly data exhibit large daily seasonality variation, relatively low weekly seasonality variation, and relatively fixed yearly seasonality variation, STLF is particularly effective for time series exhibiting yearly seasonal patterns. As such, STLF has an advantage in forecasting long-sequence time series. However, the remainder of STL is somewhat large, indicating that there may be additional factors not accounted for in STLF, such as calendar effects or special events [44].

#### Hybrid Models

The hybrid model integrating STLF and NNAR has the enhanced ability to capture the multiseasonal and nonlinear characteristics of time series and thus improved forecast accuracy across various forecast horizons. This can explain why the hybrid NNAR-STLF model exhibits the best overall performance among all the models. By contrast, the hybrid SARIMA-NNAR-STLF model exhibits a strengthened autocorrelation between its components and the daily seasonal lag owing to the single-season cycle limitation imposed by the SARIMA component. As a result, the prediction accuracy gap between hybrid NNAR-STLF and hybrid SARIMA-NNAR-STLF widens with increasing forecast horizon, although the inclusion of NNAR does result in a slight improvement in prediction accuracy. The hybrid SARIMA-NNAR model, which lacks the ability to handle multiseasonal patterns, exhibits a distinct disadvantage in medium- to long-term forecasting, with the poorest performance in both cases.

#### Implication of Errors on Real-World Applications

Inaccurate forecasts can impede effective hospital management and even interfere with decision-making processes. For example, if predicted visits are significantly lower or higher than the actual number, this can result in either inadequate or redundant allocation of personnel, consumables, and facilities. The former can negatively impact the patient’s treatment experience through long wait times, overcrowding, and resource shortage, whereas the latter can lead to resource waste and increased operating cost for the hospital.

### Limitations and Future Prospects

In this study, we used individual time-series models and their combinations to forecast hourly visits to fever clinics in children’s hospitals. Although the optimal hybrid NNAR-STLF model was able to capture the multiseasonality and nonlinear characteristics present in the time-series data, it still produced large errors in forecasting certain months owing to unaccounted external factors. Figure 15 displays the RMSE and MAE box plots for the forecast results from all the models across different months. Statistically, forecast errors were the largest in January and February, with suboptimal errors also observed in March and April. This may be attributed to the influence of the Chinese New Year, which typically falls in January or February. According to Chinese tradition, economic and social activities throughout the community are affected in the months before and after the festival.

Furthermore, the relaxation of COVID-19 containment policies in December of the previous year may have also impacted fever clinic visits. As January was the peak of the first wave of positive COVID-19 cases following the policy adjustment, data after this month lacked sufficient historical training samples, resulting in decreased prediction accuracy after January 2023. Despite this, the continued prevalence of respiratory epidemics in the postepidemic era has led to the retention of high levels of fever clinic visits, because of which fever clinic visit forecasting is still of great significance for clinical decision-making.

In future work, we will account for the effects of moving holidays and disruptive events, as the incorporation of external variables may improve the forecast accuracy [45]. The data set used in this study was obtained from a prominent provincial public children’s hospital in the Yangtze River Delta region of China. Our findings have implications for large hospitals in other regions that have established 24-hour fever clinics. However, to enhance the generalizability of our model, we will incorporate fever clinic visit data from additional medical institutions and construct high-quality, multicenter data sets for model training. Furthermore, this study used a naive averaging strategy to integrate the hybrid model results. The development of more effective fusion strategies represents another important direction for future research.

### Conclusions

In this study, we investigated the problem of visit forecasting in a fever clinic in a large public children’s hospital in China. Given the changes in clinics’ operational mode and patient visitation patterns following the outbreak of the COVID-19 epidemic, developing new forecasting models is essential for supporting intelligent hospital management. The retrospective data on hourly visits to the fever clinic can be characterized as a long-sequence time series in high frequency, with distinct temporal patterns and statistical characteristics inherent to pediatric clinics. Therefore, to identify appropriate models that accurately fit the data and exhibit robust generalization for practical management, we conducted an exploratory data analysis to reveal the seasonality and structural properties of the time-series data. On the basis of these results, we validated an ensemble of time-series models, including individual models and their combinations. We cross-validated their accuracy performance across different forecast horizons. The hybrid NNAR-STLF model was identified as the optimal model for our problem because of its ability to fit multiseasonal patterns and nonlinearity in the time-series data. Its strong performance across different forecast horizons, as indicated by the cross-validation results, further demonstrates its robustness for multiseasonal time series. The model identified in this study is applicable to hospitals with similar outpatient configurations or time series characterized as long sequence in high frequency. We also provided a new research paradigm for other time-series studies, that is, conducting an exploratory analysis revealing data characteristics before model development. However, the existing models do not account for the effects of exogenous variables, such as moving holidays and disruptive events. Future work will explore more comprehensive methods for incorporating external variables and other factors (eg, temperature, humidity, and pollutant levels) into the models to improve their prediction accuracy.

## Appendix

Multimedia Appendix 1 Autoregressive integrated moving average (ARIMA) and seasonal autoregressive integrated moving average (SARIMA) models.

## Abbreviations

ACF: autocorrelation function
ARIMA: autoregressive integrated moving average
EMR: electronic medical record
LSTM: long short-term memory
MA: moving average
MAE: mean absolute error
NNAR: neural network autoregressive
PACF: partial autocorrelation function
RMSE: root mean squared error
SARIMA: seasonal autoregressive integrated moving average
SNaive: seasonal naive
STL: seasonal-trend decomposition using Loess
STLF: seasonal and trend decomposition using Loess forecasting

## Data Availability

All the data used in this study were obtained from the electronic medical record of the fever clinic at the Children's Hospital of Zhejiang University School of Medicine. The raw electronic medical record data cannot be shared publicly because of the regulatory and legal restrictions imposed by the hospital. However, the data sets generated during this study are available from the corresponding author upon reasonable request, and the code used by this study is public in the GitHub repository [46].
