# Supplementary material for: Analyzing and Forecasting Pediatric Fever Clinic Visits in High Frequency Using Ensemble Time-Series Methods After the COVID-19 Pandemic in Hangzhou, China: Retrospective Study
Source: JMIR Med Inform. 2023 Sep 20;11:e45846. doi: 10.2196/45846 (PMC10551790; doi:10.2196/45846)
Supplement: Multimedia Appendix 1 [file medinform_v11i1e45846_app1.doc]

**Multimedia Appendix 1**

**ARIMA and SARIMA models**

The ARIMA model is denoted as $\text{ARIMA(p,d,q)}$, in which *p* is order of the AR part, *d* is degree of first differencing involved, and *q* is order of the MA part. It can be expressed in backshift notation as

$\left(1-\varphi_{1}B-\cdots-\varphi_{p}B^{p}\right)\left(1-B\right)^{d}y_{t}=c+\left(1+\theta_{1}B+\cdots+\theta_{q}B^{q}\right)\varepsilon_{t}$,

where $y_{t}$ is the value of the time series at time *t* , $\varepsilon_{t}$ denotes white noise, *c* denotes a constant, *B* is the backward shift operator, *n* applications of *B* have the effect of shifting the data back *n* periods, that is $B^{n}y_{t}=y_{t-n}$ and $B^{n}\varepsilon_{t}=\varepsilon_{t-n}$, and *B* operator can be treated using ordinary algebraic rules. $\left(1-B\right)^{d}y_{t}$ means a dth-order difference on $y_{t}$. Also, $1-\varphi_{1}B-\cdots-\varphi_{p}B^{p}$ represents the AR(p) operation and $1+\theta_{1}B+\cdots+\theta_{q}B^{q}$ represents the MA(q) operation.

The SARIMA model is denoted as $\text{ARIMA}\left(\text{p,d,q}\right)\left(\text{P,D,Q}\right)_{\text{m}}$, in which the $\text{(p,d,q)}$ represents the non-seasonal part of the model, also $\text{(P,D,Q)}_{\text{m}}$ represents the seasonal part of the model where *m* means the seasonal period. It can be written as

$\begin{matrix} \left(1-\varphi_{1}B-\cdots-\varphi_{p}B^{p}\right)\left(1-\Phi_{1}B^{m}-\cdots-\Phi_{P}B^{Pm}\right)\left(1-B\right)^{d}\left(1-B^{m}\right)^{D}y_{t}= \\ c+\left(1+\theta_{1}B+\cdots+\theta_{q}B^{q}\right)\left(1+\Theta_{1}B^{m}+\cdots+\Theta_{Q}B^{Qm}\right)\varepsilon_{t} \end{matrix}$.

Here, the non-seasonal part is similar to ARIMA model mentioned above. Besides, $\left(1-B^{\text{m}}\right)^{D}$ is the seasonal difference part, $1-\Phi_{1}B^{m}-\cdots-\Phi_{P}B^{Pm}$ is the seasonal AR part and $1+\Theta_{1}B^{m}+\cdots+\Theta_{Q}B^{Qm}$ is the seasonal MA part.
